# Vitamin D and Serum Immunoglobulin E Levels in Allergic Rhinitis: A Case-control Study from Pakistan

**DOI:** 10.7759/cureus.6495

**Published:** 2019-12-28

**Authors:** Sheeba F Ansari, Mubeen Memon, Naveed Brohi, Besham Kumar

**Affiliations:** 1 Internal Medicine, Liaquat University of Medical and Health Sciences, Jamshoro, PAK; 2 Pulmonology, Civil Hospital, Jamshoro, PAK; 3 Pulmonology, Jinnah Postgraduate Medical Centre, Karachi, PAK; 4 Internal Medicine, Jinnah Postgraduate Medical Centre, Karachi, PAK

**Keywords:** allergic rhinitis, vitamin d deficiency, vitamin d3, immunomodulation, pakistan

## Abstract

Introduction

Allergic rhinitis (AR) is the most common non-infectious rhinitis and is associated with sneezing, cough, and flu-like symptoms. The exact pathophysiology of AR remains uncertain. The deficiency of vitamin D_3_ has been documented as a probable cause of allergic conditions due to its role in immunomodulation. The aim of this study was to evaluate the role of vitamin D_3_ deficiency in allergic rhinitis.

Methods

This case-control study was conducted with 50 patients of AR and 50 healthy individuals. Serum immunoglobulin (Ig) E and vitamin D_3_ levels were measured in all study participants. Data were analyzed using SPSS v. 21.0 (IBM Corp., Armonk, NY).

Results

Mean serum IgE levels in the AR group were 553.5 ± 53.9 IU/L as compared to 219.4 ± 32.1 IU/L in the control group (p <0.0001). AR patients had mean serum vitamin D levels of 14.8 ± 7.4 ng/mL as compared to 19.1 ± 6.6 ng/mL in the control group (p=0.002). Only 10% of participants in the AR group had adequate serum vitamin D levels as compared to 26% in the controls (p=0.08).

Conclusion

Vitamin D deficiency was present in both study groups. The AR group had significantly lower mean levels of serum vitamin D than the control group. However, upon stratification, the differences were insignificant.

## Introduction

Allergic rhinitis (AR) is the most common non-infectious rhinitis globally and is associated with sneezing, cough, and flu-like symptoms. It is an immune-mediated phenomenon and is initiated when the allergen is exposed to the nasal mucosa, which potentiates an immunoglobulin E (IgE)-mediated inflammatory cascade [1]. It plays a significant role in debilitating general health and everyday routine. AR affects school attendance, workplace performance, and social life [1]. Even after the profound impact on the quality of life and healthcare economics, there hasn’t been much control over the condition, which is due to its unclear pathogenesis [1].

The etiology of AR remains an active area of medical research. Vitamin D_3_ deficiency has been researched and documented as a probable causative factor in allergic conditions such as food allergies [2], asthma [3], and eczema [4]. Some researchers have also associated vitamin D_3_ deficiency as a disease-causing or disease-modifying factor in AR [5]. The activity of vitamin D_3_ has a profound impact on innate and adaptive immunity. It influences the activity of various cells of the immunity system, such as B cells, T cells, macrophages, and monocytes. It also modulates immunity by influencing the activity of various cytokines and Igs, which mediate allergic disorders. Keeping in view the essential role of vitamin D_3_ in immunomodulation, researchers focused their efforts on studying the role of vitamin D_3_ deficiency in allergic conditions and its supplementation in susceptible individuals has been regarded as a protective measure against AR [6].

In Pakistan, as many as 78% of individuals have insufficient to deficient levels of vitamin D [7]. Studies have been conducted to assess the role of vitamin D_3_ deficiency in bronchial asthma and its supplementation in susceptible individuals in improving respiratory parameters in these participants [8-9]. However, to the best of our knowledge, the role of vitamin D_3_ deficiency has not been studied in allergic rhinitis patients. Hence, we conducted this study.

## Materials and methods

This case-control study was conducted in the department of respiratory diseases of Civil Hospital, Pakistan, after attaining ethical approval from the institutional review board. The study was conducted from July 1 to September 30, 2018. Fifty patients diagnosed with AR were included in the “cases” group and their 50 healthy counterparts were regarded as the “controls” group after attaining informed consent. Controls were selected from the attendants of these patients to maintain socio-demographic equity. AR was defined as per the recommendations of the American Academy of Otolaryngology-Head and Neck Surgery and the American Academy of Family Physicians. According to this recommendation, the diagnosis of allergic rhinitis is based on history and physical findings consistent with an allergic cause. The manifestations can include clear rhinorrhea, pale nasal mucosal layer, red watery eyes, along with nasal congestion, runny or itchy nose, and sneezing [10].

For all study participants, demographic characteristics included in the study were age, gender, residence (rural/urban), and occupation (indoor/outdoor) were recorded. Clinical characteristics, including height and weight, were recorded in meters and kilograms respectively. Body mass index (BMI) was calculated as weight divided by height squared. BMI was classified as normal or underweight (<23 kg/m2), overweight (23-<25 kg/m2), and obese (≥25 kg/m2) as evident by the World Health Organization (WHO) classification [11]. Serum IgE levels were obtained for all participants. For IgE levels, a 1470 Wizard gamma-counter (PerkinElmer, Finland) along with ImmunoCAP 100 (Phadia, Sweden) were utilized. IgE levels >150 IU/mL were regarded as elevated [12]. Serum 25-hydroxyvitamin D (25(OH)D) was measured to determine vitamin D deficiency. 25(OH)D <20 ng/mL were taken as vitamin D deficiency, 20-30 ng/mL indicated vitamin D insufficiency, and 25(OH)D > 50 ng/mL showed optimal levels [13].

For biostatistical analysis, SPSS version 21.0 (IBM Corp., Armonk, NY) was utilized. For continuous variables, mean and standard deviation (SD) were calculated and the independent T-test was used for comparison. For stratified variables, frequencies and percentages were calculated and chi-square was used for comparison. A p-value of ≤0.05 was considered statistically significant.

## Results

The study sample comprised 100 patients - 50 cases and 50 controls. Men were more common in both study groups (76% in cases and 86% in controls). Most of the study samples were dwelling in the rural area (n=71; 71%) and did outdoor jobs (n=65; 65%). Cases were younger in age with a mean age of 26.5 ± 12.6 years and controls had a mean age of 28.2 ± 11.3 years (p=0.4). The control group was slightly overweight with a mean BMI of 24.1 ± 6.2 kg/m^2^, and the cases group had a mean BMI of 27.3 ± 5.7 kg/m^2^ (p=0.008).

The participants in the cases group suffering from allergic rhinitis had elevated mean levels of serum IgE as compared to the controls (553.5 ± 53.9 vs. 219.4 ± 32.1 IU/L; p <0.0001). When serum vitamin D levels were compared for both study groups, it was seen that patients of AR had mean serum vitamin D levels of 14.8 ± 7.4 ng/mL as compared to 19.1 ± 6.6. The differences were statistically significant (p=0.002). However, as seen in Table 1, when serum vitamin D levels were stratified into adequate, inadequate, and insufficient, the differences were insignificant between the two study groups (p=0.08). The comparison of all other stratified variables is shown in Table 1.

**Table 1:** Comparison of the characteristics of allergic rhinitis patients and their healthy counterparts in the study IgE: immunoglobulin E

Patient Parameters	Cases (n=50)	Controls (n=50)	p-value
Age, years			
18-40	27 (54%)	24 (48%)	0.54
> 40	23 (46%)	26 (52%)	
Gender			
Male	38 (76%)	43 (86%)	0.20
Female	12 (24%)	7 (14%)	
Region of residence			
Urban	16 (32%)	13 (26%)	0.50
Rural	34 (68%)	37 (74%)	
Occupation			
Indoor jobs	11 (22%)	24 (48%)	0.006
Outdoor jobs	39 (78%)	26 (52%)	
Body mass index, kg/m^2^			
Normal or less (<23)	13 (26%)	25 (50%)	0.04
Overweight (23–<25)	23 (46%)	14 (28%)	
Obese (≥25)	14 (28%)	11 (22%)	
Serum IgE, IU/L			
Normal	12 (24%)	44 (88%)	<0.00001
Raised	38 (76%)	6 (12%)	
Serum 25-hydroxyvitamin D, ng/mL			
Adequate (>50)	5 (10%)	13 (26%)	0.08
Inadequate (20-30)	33 (66%)	30 (60%)	
Deficient (<20)	12 (24%)	7 (14%)	

## Discussion

In this study, both groups had deficient levels of vitamin D. The study group with allergic rhinitis had significantly lower mean levels of serum vitamin D as compared to the control group. However, upon stratification, the results were weakly insignificant.

IgE, which mediates allergic immune responses, has been shown to have an inverse relationship with serum vitamin D levels. Patients with low levels of vitamin D have high levels of IgE [14]. IgE is the basis of all allergic responses. There have been various reports of AR in children and adults with low levels of vitamin D [15-17]. Similarly, there have been reports of antenatal maternal vitamin D deficiency with a higher incidence of atopy in newborns [18]. The Nord-Trøndelag Health Study (HUNT) was a longitudinal cohort conducted in Norway. It collected serum vitamin D levels at baseline and then followed the individuals for around 11 years. In men, 9% developed AR; the adjusted odds ratio (AOR) was 2.55 at vitamin D <50nmol/L. In women, 15% developed AR; however, the AOR was 0.83 for each 25 nmol/L reduction in vitamin D levels [19]. Hence, another hypothesis came forward that there might be different impacts of vitamin D on AR development in men and women.

As with our study, there have been other studies in the literature, which showed little to no impact of vitamin D deficiency on the development or worsening or AR. In a study with Turkish children, mean vitamin D levels in the AR group were 18.07 ± 6.1 ng/mL, as compared to 14.81 ± 4.86 ng/mL in the non-allergic rhinitis (NAR) group, and 24.03 ± 9.43 ng/mL in control group (p=0.001). More children in NAR were vitamin D deficient as compared to AR 67% vs. 89%). Vitamin D levels did not statistically correlate to allergen sensitivity and AR duration and severity [20]. Similarly, in a Korean study group, mean serum vitamin D levels were significantly lower in patients with atopic dermatitis but not asthma, AR, or IgE sensitization [21].

Some interventional studies have highlighted the role of supplementation of vitamin D in the alleviation of AR symptoms. In Heine et al., vitamin D supplementation in vitamin D deficient mice resulted in immunomodulation, which favored protection against allergic triggers. Production of pro-inflammatory cytokines was reduced and that of anti-inflammatory cytokines was increased by vitamin D supplementation [22]. Jerzynska et al. supplemented children with vitamin D during the pollen season and observed fewer manifestations of AR as compared to the placebo group [23].

The study has its limitations. Firstly, the sample size was small. A large proportion of the study sample was outdoors. which was thought to bring a bias on higher vitamin D levels to the study (due to sun exposure); however, most participants in both study groups had lower vitamin D levels. We recommend further studies with the control group selected on the basis of blood analysis and eliminating individuals with low vitamin D levels. This will bring more strength to the study mythology, however, it was not possible in our case due to limited resources.

## Conclusions

Our study showed low levels of vitamin D in both the cases and the controls groups; however, cases had significantly lower levels. Upon stratification, the differences were weakly insignificant. The results of this study are not concrete and conclusive, however, it is the first of its kind from this region. It puts forward the need for more robust clinical trials to explain the association of serum vitamin D levels and allergic rhinitis in the Pakistani population.
